# Supplementary material for: Modelling the impact of migrants on the success of the HIV care and treatment program in Botswana
Source: PLoS One. 2020 Jan 15;15(1):e0226422. doi: 10.1371/journal.pone.0226422 (PMC6961860; doi:10.1371/journal.pone.0226422)
Supplement: S1 Table — Full data inputs for the model with justifications where applicable. (DOCX) [file pone.0226422.s001.docx]

# S1 Table: Botswana data inputs

| **Indicator** | **Value [Reference]** | |
| --- | --- | --- |
|  | **Citizens** | **Immigrants** |
| Population size*^£^*  We assumed that 70% of the total population were aged 15-65 years old. | 1,604,400 (2014) [23] | 112,195 (2016) [13] |
| Birth rate (births per woman per year)  Botswana's fertility rate is 2.83 and assumed to be 0.05 live births per woman in the 15-65 year population and we assumed the rate to be similar among immigrants. | 0.05* | 0.05* |
| Estimated HIV prevalence (%)*^£^* | 19.5 (2017) [12, 19] | 15% (2017) [14] |
| HIV testing  We assumed a lower testing rate among immigrants due to lack of access. | 31–55% tested (2017) [24] | 35%^*^ |
| Number of HIV diagnoses per year  We projected that 1,300 immigrants were new HIV diagnoses in 2017 based on projections from the 15% who were HIV positive in the BCPP [14]. | 2010: 13,000; 2013: 9,100; 2016: 10,000; 2017: 14,000 [24] | 1,300* |
| PLHIV aware of their status (%) | 86% (83–89%) [12, 20, 24] | 25% [14] |
| Average time taken to be linked to care (years)  Average time to link was based on 3 months for citizens and much longer for immigrants. This would be much longer for those with lower CD4+ counts. | 0.3* | 0.9* |
| Average time taken to be linked to care for people with CD4<200 (years)  The time was assumed to be the same for citizens and immigrants because such patients usually have AIDS defining illnesses and will be forced to seek medical care anyway irrespective of migration status. | 0.5* | 0.5* |
| Diagnosed PLHIV in care (%)  A higher proportion was assumed for citizens based on 2017 but much lower for immigrants. | 90%* | 75* |
| Total number of people on treatment  The total number of immigrants on treatment was computed based on 15% assumed HIV prevalence inflated to account for underestimations. | 380,000 [12] | 18,600* |
| PLHIV in care on treatment (%) | 84–90% [12, 20] | 76% [14] |
| Treatment failure rate (%)  This was based on an assumption that 10% of PLHIV on treatment in Botswana had failed 1^st^ line ART regimen, and expected to be higher in immigrants. | 7%* | 25%* |
| Percentage of people in care who are lost to follow-up per year (%/year).  Botswana experiences a low lost to follow-up rate but expected to be much higher for immigrants. This would be much lower for PLHIV who are very sick with CD4<200 below. | 5%* | 15%* |
| Percentage of people with CD4<200 lost to follow-up (%/year) | 4%* | 5%% |
| Viral load monitoring (number/year)  Viral load monitoring was based on the number in care on treatment. | 335,000* | 11,000* |
| People on ART with viral suppression (%) | 95% [12, 20] | 82% [25] |
| Proportion of those with VL failure who are provided with effective adherence support or a successful new regimen (%/year) | 90%* | 60* |
| Pregnant women on PMTCT (%) | 2015: 92%; 2016: 95% [24] | 60%* |
| Number of women on PMTCT (Option B/B+) | 120,000 [24] | 500* |
| Percentage of HIV-positive women who breastfeed | 90%[24] | 90* |
| Percentage of people covered by ARV-based prophylaxis | 0.5%* | 0%* |
| Average number of acts with regular partners per person per year | 54* | 54* |
| Average number of acts with casual partners per person per year | 30* | 30* |
| Average number of acts with commercial partners per person per year | 15* | 15* |
| Percentage of people who used a condom at last act with regular partners | 82% [24] | 82%* |
| Percentage of males who have been circumcised | 45% [19] | 30%* |
| Number of HIV-related deaths | 14,000 in 2005 and 3,900 in 2016 [12, 24, 26] | 1,500*; 8% of PLHIV on ART [25] |
| Number of people initiating ART each year | 5000* | 500* |
| Interaction-related transmissibility (% per act) |  |  |
| Insertive penile-vaginal intercourse | 0.04–0.14% |  |
| Receptive penile-vaginal intercourse | 0.08–0.11% |  |
| Insertive penile-anal intercourse | 0.11–0.28% |  |
| Receptive penile-anal intercourse | 1.38–1.86% |  |
| Intravenous injection | 0.80–2.40% |  |
| Mother-to-child (breastfeeding) | 36.70–44.00% |  |
| Mother-to-child (non-breastfeeding) | 20.50–27.00% |  |
| Condom use | 95–98% |  |
| Circumcision | 58–67% |  |
| Diagnosis behaviour change | 0–68% |  |
| STI cofactor increase | 265–519% |  |
| Opiate substitution therapy | 54–68% |  |
| PMTCT | 90–93% |  |
| ARV-based prophylaxis | 73–80% |  |
| Efficacy of unsuppressive ART | 50–80% |  |
| Efficacy of suppressive ART | 92–100% |  |

*^£^A factor was considered to represent the targeted age group (15–65 years); *Assumption in 2017; ARV: antiretroviral; ART: antiretroviral therapy; PLHIV: people living with HIV; PMTCT: prevention of mother-to-child transmission; STI: sexually transmitted infections.*
