# Supplementary material for: Modelling the impact of migrants on the success of the HIV care and treatment program in Botswana
Source: PLoS One. 2020 Jan 15;15(1):e0226422. doi: 10.1371/journal.pone.0226422 (PMC6961860; doi:10.1371/journal.pone.0226422)
Supplement: S2 Table — Full set of parameters used to fit the model. (DOCX) [file pone.0226422.s002.docx]

# S2 Table: Model fitting

| Initial HIV prevalence: Immigrants | 0.15 |
| --- | --- |
| Initial HIV prevalence: Citizen | 0.176 |
| Force-of-infection: Immigrants | 1.1786471 |
| Force-of-infection: Citizen | 1.8030469 |
| Inhomogeneity: Immigrants | 0 |
| Inhomogeneity: Citizen | 0 |
| Population size: Immigrants | 112784 |
| Population size: Citizen | 1384540 |
| Relative death rate for populations (unit-less): Immigrants | 1 |
| Relative death rate for populations (unit-less): Citizen | 1 |
| Year to fix PLHIV aware of their status | 2100 |
| Year to fix diagnosed PLHIV in care | 2100 |
| Year to fix PLHIV in care on treatment | 2017 |
| Year to fix people on ART with viral suppression | 2017 |
| Year to fix pregnant women and mothers on PMTCT | 2100 |
| Normalization factor for transmissibility | 0.43 |
| Male-female insertive transmissibility (per act) | 0.0004 |
| Male-female receptive transmissibility (per act) | 0.0008 |
| Male-male insertive transmissibility (per act) | 0.0011 |
| Male-male receptive transmissibility (per act) | 0.0138 |
| Injection-related transmissibility (per injection) | 0.008 |
| Mother-to-child breastfeeding transmissibility | 0.367 |
| Mother-to-child no-breastfeeding transmissibility | 0.205 |
| Relative transmissibility for acute HIV (unit-less) | 5.6 |
| Relative transmissibility for CD4>500 (unit-less) | 1 |
| Relative transmissibility for CD4>350 (unit-less) | 1 |
| Relative transmissibility for CD4>200 (unit-less) | 1 |
| Relative transmissibility for CD4>50 (unit-less) | 3.49 |
| Relative transmissibility for CD4<50 (unit-less) | 7.17 |
| Relative transmissibility with STIs (unit-less) | 2.65 |
| Progression time from acute HIV (years) | 0.24 |
| Progression from CD4>500 (years) | 0.95 |
| Progression from CD4>350 (years) | 3 |
| Progression from CD4>200 (years) | 2.74 |
| Progression from CD4>50 (years) | 1.5 |
| Treatment recovery into CD4>500 (years) | 2.2 |
| Treatment recovery into CD4>350 (years) | 1.42 |
| Treatment recovery into CD4>200 (years) | 2.14 |
| Treatment recovery into CD4>50 (years) | 0.66 |
| Time after initiating ART to achieve viral suppression (years) | 0.2 |
| Number of VL tests recommended per person per yea | 1 |
| Death rate for AIDS (CD4>50) (per year) | 0.059 |
| Death rate for AIDS (CD4<50) (per year) | 0.323 |
| Relative death rate on suppressive ART (unit-less) | 0.23 |
| Relative death rate on unsuppressive ART (unit-less) | 0.4878 |
| Efficacy of unsuppressive ART | 0.5 |
| Efficacy of suppressive ART | 1.0 |
| Efficacy of PMTCT | 0.9 |
| Efficacy of ARV-based prophylaxis | 0.73 |
| Efficacy of condoms | 0.95 |
| Efficacy of circumcision | 0.58 |
| Efficacy of diagnosis for behaviour change | 0 |

*ART: antiretroviral therapy; ARV: antiretroviral; PMTCT: prevention of mother to child transmission; VL: viral load; AIDS: acquired immunodeficiency syndrome; STI: sexually transmitted infection; PLHIV: people living with HIV*
