# Supplementary material for: Modelling the impact of migrants on the success of the HIV care and treatment program in Botswana
Source: PLoS One. 2020 Jan 15;15(1):e0226422. doi: 10.1371/journal.pone.0226422 (PMC6961860; doi:10.1371/journal.pone.0226422)
Supplement: S3 Table — Table shows the full results for the baseline scenario. Variables include: new HIV infections, HIV related deaths, number of people living with HIV (PLHIV), prevalence and incidence for immigrants and citizens. (DOCX) [file pone.0226422.s003.docx]

# S3 Table: Baseline scenario results (2010–30)

| **Indicator** | **Pop** | **2010** | **2011** | **2012** | **2013** | **2014** | **2015** | **2016** | **2017** | **2018** | **2019** | **2020** | **2021** | **2022** | **2023** | **2024** | **2025** | **2026** | **2027** | **2028** | **2029** | **2030** |
| --- | --- | --- | --- | --- | --- | --- | --- | --- | --- | --- | --- | --- | --- | --- | --- | --- | --- | --- | --- | --- | --- | --- |
| New HIV infections | Total | 31271 | 13506 | 14685 | 16167 | 12861 | 12221 | 12178 | 12254 | 12439 | 12697 | 13015 | 13393 | 13826 | 14308 | 14835 | 15404 | 16011 | 16656 | 17338 | 18057 | 18813 |
| New HIV infections | Imm | 1539 | 682 | 746 | 824 | 677 | 655 | 658 | 665 | 677 | 692 | 709 | 728 | 750 | 774 | 800 | 828 | 858 | 890 | 924 | 959 | 996 |
| New HIV infections | Cit | 29733 | 12824 | 13939 | 15343 | 12183 | 11566 | 11520 | 11589 | 11762 | 12005 | 12306 | 12665 | 13076 | 13534 | 14035 | 14575 | 15153 | 15766 | 16414 | 17098 | 17817 |
| HIV-related deaths | Total | 7,399 | 2,382 | 1,604 | 1,298 | 1,043 | 929 | 853 | 799 | 764 | 740 | 725 | 718 | 718 | 724 | 734 | 747 | 764 | 783 | 804 | 827 | 852 |
| HIV-related deaths | Imm | 480 | 165 | 120 | 103 | 90 | 85 | 83 | 82 | 81 | 81 | 80 | 80 | 81 | 81 | 82 | 83 | 85 | 86 | 88 | 90 | 92 |
| HIV-related deaths | Cit. | 6,918 | 2,217 | 1,484 | 1,195 | 953 | 843 | 770 | 718 | 683 | 659 | 644 | 637 | 637 | 642 | 652 | 664 | 679 | 697 | 716 | 737 | 759 |
| PLHIV | Total | 260597 | 272643 | 282261 | 293872 | 305449 | 314419 | 323133 | 331853 | 340666 | 349656 | 358875 | 368372 | 378196 | 388391 | 398994 | 410043 | 421570 | 433607 | 446185 | 459334 | 473085 |
| PLHIV | Imm | 16918 | 17381 | 17766 | 18258 | 18757 | 19143 | 19522 | 19904 | 20291 | 20689 | 21098 | 21522 | 21961 | 22418 | 22895 | 23393 | 23913 | 24458 | 25028 | 25624 | 26248 |
| PLHIV | Cit | 243679 | 255262 | 264495 | 275614 | 286691 | 295276 | 303611 | 311949 | 320374 | 328967 | 337776 | 346851 | 356235 | 365973 | 376099 | 386650 | 397657 | 409149 | 421157 | 433710 | 446837 |
| HIV prevalence (%) | Total | 17% | 17% | 17% | 17% | 17% | 17% | 17% | 16% | 16% | 16% | 16% | 15% | 15% | 15% | 15% | 14% | 14% | 14% | 14% | 13% | 13% |
| HIV prevalence (%) | Imm | 15% | 15% | 15% | 14% | 14% | 14% | 14% | 13% | 13% | 13% | 12% | 12% | 12% | 12% | 11% | 11% | 11% | 11% | 10% | 10% | 10% |
| HIV prevalence (%) | Cit | 18% | 18% | 18% | 17% | 17% | 17% | 17% | 17% | 16% | 16% | 16% | 16% | 15% | 15% | 15% | 15% | 14% | 14% | 14% | 14% | 14% |
| HIV incidence (/100 py) | Total | 2.53 | 1.05 | 1.09 | 1.14 | 0.87 | 0.79 | 0.75 | 0.72 | 0.70 | 0.68 | 0.67 | 0.65 | 0.65 | 0.64 | 0.63 | 0.63 | 0.62 | 0.62 | 0.61 | 0.61 | 0.61 |
| HIV incidence (/100 py) | Imm | 1.61 | 0.68 | 0.71 | 0.75 | 0.59 | 0.55 | 0.53 | 0.51 | 0.49 | 0.48 | 0.47 | 0.46 | 0.46 | 0.45 | 0.44 | 0.44 | 0.44 | 0.43 | 0.43 | 0.43 | 0.42 |
| HIV incidence (/100 py) | Cit | 2.61 | 1.08 | 1.12 | 1.18 | 0.89 | 0.81 | 0.77 | 0.74 | 0.72 | 0.70 | 0.68 | 0.67 | 0.66 | 0.65 | 0.65 | 0.64 | 0.64 | 0.63 | 0.63 | 0.63 | 0.62 |

*PLHIV: people living with HIV; infect. = infections; Imm = immigrants; Cit = citizens; py: person-*
