# Supplementary material for: Modelling the impact of migrants on the success of the HIV care and treatment program in Botswana
Source: PLoS One. 2020 Jan 15;15(1):e0226422. doi: 10.1371/journal.pone.0226422 (PMC6961860; doi:10.1371/journal.pone.0226422)
Supplement: S4 Table — Table shows the full results for the scenario when scaling up in citizens only. Variables include: new HIV infections, HIV related deaths, number of people living with HIV (PLHIV), prevalence and incidence for immigrants and citizens. (DOCX) [file pone.0226422.s004.docx]

**S4 Table: Scaling up to 95-95-95 in citizens only**

| **Indicator** | **Pop** | **2010** | **2011** | **2012** | **2013** | **2014** | **2015** | **2016** | **2017** | **2018** | **2019** | **2020** | **2021** | **2022** | **2023** | **2024** | **2025** | **2026** | **2027** | **2028** | **2029** | **2030** |
| --- | --- | --- | --- | --- | --- | --- | --- | --- | --- | --- | --- | --- | --- | --- | --- | --- | --- | --- | --- | --- | --- | --- |
| **New HIV infections** | **Total** | 31,271 | 13,506 | 14,685 | 16,167 | 12,861 | 12,221 | 12,178 | 12,254 | 12,439 | 12,676 | 12,683 | 12,497 | 12,223 | 11,894 | 11,539 | 11,181 | 10,837 | 10,517 | 10,229 | 9,976 | 9,766 |
| **New HIV infections** | **Imm** | 1,539 | 682 | 746 | 824 | 677 | 655 | 658 | 665 | 677 | 689 | 681 | 666 | 650 | 632 | 614 | 595 | 577 | 561 | 546 | 532 | 521 |
| **New HIV infections** | **Cit** | 29,733 | 12,824 | 13,939 | 15,343 | 12,183 | 11,566 | 11,520 | 11,589 | 11,762 | 11,987 | 12,002 | 11,831 | 11,573 | 11,262 | 10,925 | 10,586 | 10,259 | 9,957 | 9,684 | 9,443 | 9,245 |
| **HIV-related deaths** | **Total** | 7,399 | 2,382 | 1,604 | 1,298 | 1,043 | 929 | 853 | 799 | 764 | 740 | 717 | 693 | 670 | 647 | 623 | 600 | 578 | 559 | 541 | 527 | 515 |
| **HIV-related deaths** | **Imm** | 480 | 165 | 120 | 103 | 90 | 85 | 83 | 82 | 81 | 81 | 78 | 76 | 75 | 74 | 74 | 73 | 73 | 72 | 72 | 71 | 71 |
| **HIV-related deaths** | **Cit** | 6,918 | 2,217 | 1,484 | 1,195 | 953 | 843 | 770 | 718 | 683 | 659 | 639 | 617 | 595 | 573 | 549 | 527 | 505 | 486 | 470 | 456 | 444 |
| **PLHIV** | **Total** | 260,597 | 272,643 | 282,261 | 293,872 | 305,449 | 314,419 | 323,133 | 331,853 | 340,666 | 349,654 | 358,761 | 367,731 | 376,427 | 384,779 | 392,748 | 400,319 | 407,498 | 414,307 | 420,774 | 426,931 | 432,814 |
| **PLHIV** | **Imm** | 16,918 | 17,381 | 17,766 | 18,258 | 18,757 | 19,143 | 19,522 | 19,904 | 20,291 | 20,689 | 21,088 | 21,473 | 21,842 | 22,190 | 22,518 | 22,824 | 23,110 | 23,376 | 23,624 | 23,856 | 24,073 |
| **PLHIV** | **Cit** | 243,679 | 255,262 | 264,495 | 275,614 | 286,691 | 295,276 | 303,611 | 311,949 | 320,374 | 328,965 | 337,673 | 346,257 | 354,585 | 362,589 | 370,230 | 377,494 | 384,388 | 390,931 | 397,150 | 403,075 | 408,740 |
| **HIV prevalence (%)** | **Total** | 17% | 17% | 17% | 17% | 17% | 17% | 17% | 16% | 16% | 16% | 16% | 15% | 15% | 15% | 14% | 14% | 14% | 13% | 13% | 13% | 12% |
| **HIV prevalence (%)** | **Imm** | 15% | 15% | 15% | 14% | 14% | 14% | 14% | 13% | 13% | 13% | 12% | 12% | 12% | 11% | 11% | 11% | 10% | 10% | 10% | 9% | 9% |
| **HIV prevalence (%)** | **Cit** | 18% | 18% | 18% | 17% | 17% | 17% | 17% | 17% | 16% | 16% | 16% | 16% | 15% | 15% | 15% | 14% | 14% | 14% | 13% | 13% | 12% |
| **HIV incidence (per 100 p.y.)** | **Total** | 2.53 | 1.05 | 1.09 | 1.14 | 0.87 | 0.79 | 0.75 | 0.72 | 0.70 | 0.68 | 0.65 | 0.61 | 0.57 | 0.53 | 0.49 | 0.45 | 0.42 | 0.39 | 0.36 | 0.33 | 0.31 |
| **HIV incidence (per 100 p.y.)** | **Imm** | 1.61 | 0.68 | 0.71 | 0.75 | 0.59 | 0.55 | 0.53 | 0.51 | 0.49 | 0.48 | 0.45 | 0.42 | 0.39 | 0.37 | 0.34 | 0.31 | 0.29 | 0.27 | 0.25 | 0.23 | 0.22 |
| **HIV incidence (per 100 p.y.)** | **Cit** | 2.61 | 1.08 | 1.12 | 1.18 | 0.89 | 0.81 | 0.77 | 0.74 | 0.72 | 0.70 | 0.67 | 0.63 | 0.58 | 0.54 | 0.50 | 0.46 | 0.43 | 0.40 | 0.37 | 0.34 | 0.32 |

*PLHIV: people living with HIV; infect. = infections; Imm = immigrants; Cit = citizens; py: person-year*
