# Supplementary material for: Modelling the impact of migrants on the success of the HIV care and treatment program in Botswana
Source: PLoS One. 2020 Jan 15;15(1):e0226422. doi: 10.1371/journal.pone.0226422 (PMC6961860; doi:10.1371/journal.pone.0226422)
Supplement: S5 Table — Table shows the full results for the scenario when scaling up in both immigrants and citizens. Variables include: new HIV infections, HIV related deaths, number of people living with HIV (PLHIV), prevalence and incidence for immigrants and citizens. (DOCX) [file pone.0226422.s005.docx]

**S5 Table: Scaling up to 95-95-95 in both citizens and immigrants**

| **Indicator** | **Pop** | **2010** | **2011** | **2012** | **2013** | **2014** | **2015** | **2016** | **2017** | **2018** | **2019** | **2020** | **2021** | **2022** | **2023** | **2024** | **2025** | **2026** | **2027** | **2028** | **2029** | **2030** |
| --- | --- | --- | --- | --- | --- | --- | --- | --- | --- | --- | --- | --- | --- | --- | --- | --- | --- | --- | --- | --- | --- | --- |
| **New HIV infections** | **Total** | 31,271 | 13,506 | 14,685 | 16,167 | 12,861 | 12,221 | 12,178 | 12,254 | 12,439 | 12,670 | 12,548 | 12,104 | 11,524 | 10,878 | 10,216 | 9,576 | 8,984 | 8,453 | 7,991 | 7,601 | 7,287 |
| **New HIV infections** | **Imm** | 1,539 | 682 | 746 | 824 | 677 | 655 | 658 | 665 | 677 | 690 | 682 | 658 | 627 | 592 | 556 | 522 | 490 | 462 | 437 | 417 | 401 |
| **New HIV infections** | **Cit** | 29,733 | 12,824 | 13,939 | 15,343 | 12,183 | 11,566 | 11,520 | 11,589 | 11,762 | 11,980 | 11,866 | 11,446 | 10,898 | 10,286 | 9,660 | 9,054 | 8,494 | 7,991 | 7,554 | 7,184 | 6,886 |
| **HIV-related deaths** | **Total** | 7,399 | 2,382 | 1,604 | 1,298 | 1,043 | 929 | 853 | 799 | 764 | 740 | 716 | 689 | 660 | 627 | 593 | 560 | 527 | 498 | 472 | 451 | 435 |
| **HIV-related deaths** | **Imm** | 480 | 165 | 120 | 103 | 90 | 85 | 83 | 82 | 81 | 81 | 79 | 76 | 72 | 67 | 62 | 56 | 50 | 45 | 40 | 36 | 33 |
| **HIV-related deaths** | **Citi** | 6,918 | 2,217 | 1,484 | 1,195 | 953 | 843 | 770 | 718 | 683 | 659 | 637 | 613 | 587 | 560 | 532 | 504 | 477 | 453 | 433 | 415 | 402 |
| **PLHIV** | **Total** | 260,597 | 272,643 | 282,261 | 293,872 | 305,449 | 314,419 | 323,133 | 331,853 | 340,666 | 349,654 | 358,718 | 367,458 | 375,652 | 383,201 | 390,069 | 396,261 | 401,815 | 406,790 | 411,251 | 415,267 | 418,909 |
| **PLHIV** | **Imm** | 16,918 | 17,381 | 17,766 | 18,258 | 18,757 | 19,143 | 19,522 | 19,904 | 20,291 | 20,689 | 21,090 | 21,473 | 21,828 | 22,151 | 22,441 | 22,698 | 22,926 | 23,126 | 23,303 | 23,460 | 23,600 |
| **PLHIV** | **Cit** | 243,679 | 255,262 | 264,495 | 275,614 | 286,691 | 295,276 | 303,611 | 311,949 | 320,374 | 328,965 | 337,628 | 345,986 | 353,824 | 361,050 | 367,628 | 373,562 | 378,890 | 383,664 | 387,948 | 391,807 | 395,309 |
| **HIV prevalence (%)** | **Total** | 17% | 17% | 17% | 17% | 17% | 17% | 17% | 16% | 16% | 16% | 16% | 15% | 15% | 15% | 14% | 14% | 13% | 13% | 13% | 12% | 12% |
| **HIV prevalence (%)** | **Imm** | 15% | 15% | 15% | 14% | 14% | 14% | 14% | 13% | 13% | 13% | 12% | 12% | 12% | 11% | 11% | 11% | 10% | 10% | 10% | 9% | 9% |
| **HIV prevalence (%)** | **Cit** | 18% | 18% | 18% | 17% | 17% | 17% | 17% | 17% | 16% | 16% | 16% | 16% | 15% | 15% | 14% | 14% | 14% | 13% | 13% | 12% | 12% |
| **HIV incidence (per 100 p.y.)** | **Total** | 2.53 | 1.05 | 1.09 | 1.14 | 0.87 | 0.79 | 0.75 | 0.72 | 0.70 | 0.68 | 0.64 | 0.59 | 0.54 | 0.48 | 0.43 | 0.39 | 0.35 | 0.31 | 0.28 | 0.25 | 0.23 |
| **HIV incidence (per 100 p.y.)** | **Imm** | 1.61 | 0.68 | 0.71 | 0.75 | 0.59 | 0.55 | 0.53 | 0.51 | 0.49 | 0.48 | 0.45 | 0.42 | 0.38 | 0.34 | 0.31 | 0.28 | 0.25 | 0.22 | 0.20 | 0.18 | 0.17 |
| **HIV incidence (per 100 p.y.)** | **Cit** | 2.61 | 1.08 | 1.12 | 1.18 | 0.89 | 0.81 | 0.77 | 0.74 | 0.72 | 0.70 | 0.66 | 0.61 | 0.55 | 0.50 | 0.44 | 0.40 | 0.35 | 0.32 | 0.29 | 0.26 | 0.24 |

*PLHIV: people living with HIV; infect. = infections; Imm = immigrants; Cit = citizens; py: person-year*
