# Supplementary figures and images for: Modelling the impact of migrants on the success of the HIV care and treatment program in Botswana
Source: PLoS One. 2020 Jan 15;15(1):e0226422. doi: 10.1371/journal.pone.0226422 (PMC6961860; doi:10.1371/journal.pone.0226422)

**S1 Fig: Calibration of the model and the fit achieved**

*
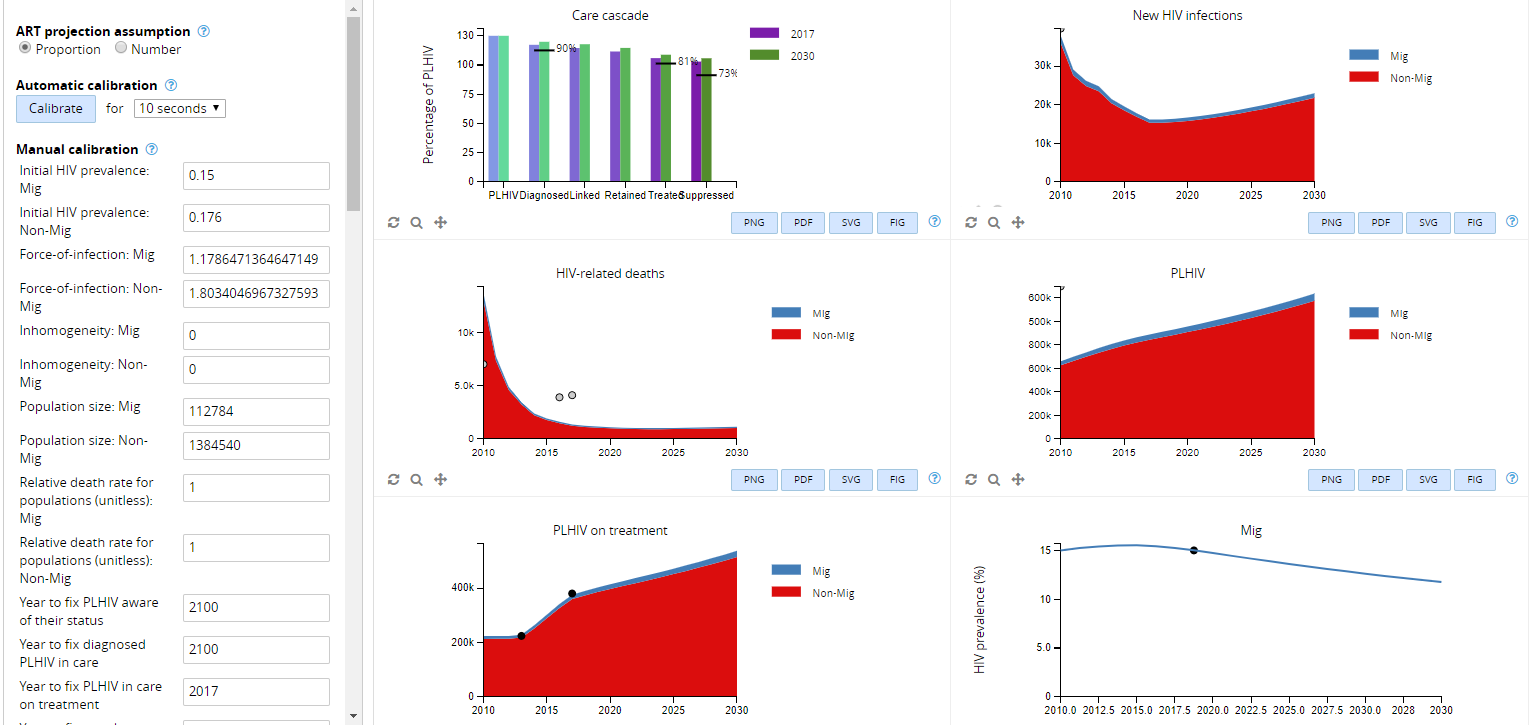
*


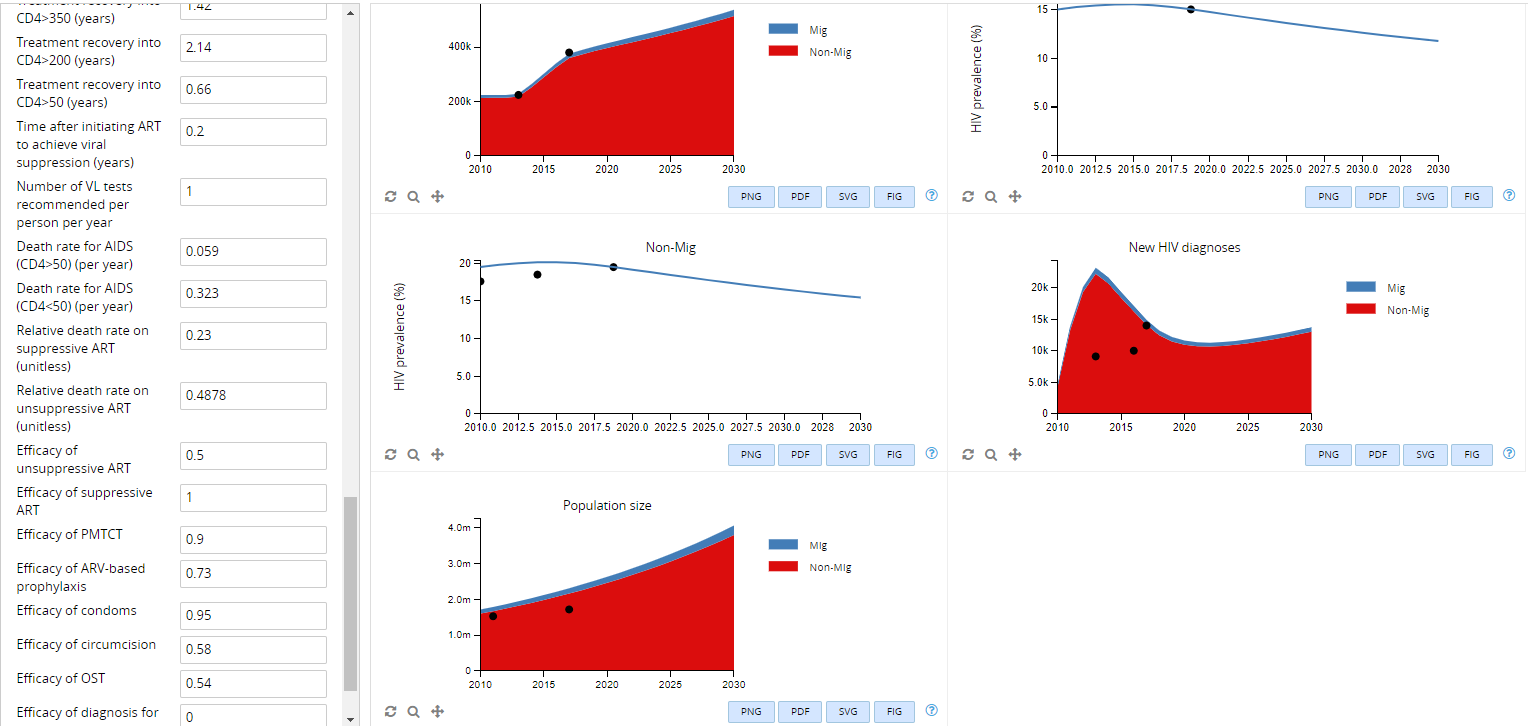

Supplement: S1 Fig — Figure shows alongside model fitting parameters the fit that was achieved for the model. (DOCX) [file pone.0226422.s006.docx]
